# Myeloid differentiation factor-2/LY96, a new predictive biomarker of metastasis in prostate cancer: Clinical implications as a potential therapeutic target

**DOI:** 10.21203/rs.3.rs-2968406/v1

**Published:** 2023-06-09

**Authors:** Marina Ferrari, Li Wang, Luke Hoeppner, Eunsil Hahm, Jindan Yu, Timothy Kuzel, ADRIAN MANSINI

**Affiliations:** University of Minnesota; Emory University; Rush University Medical Center

**Keywords:** MD2, Prostate Cancer, CRPC, biomarker

## Abstract

Relapsed prostate cancer (CaP), usually treated with androgen deprivation therapy, acquires resistance to develop into lethal metastatic castration-resistant CaP. The cause of resistance remains elusive, and the lack of biomarkers predictive of castration-resistance emergence is a stumbling block in managing the disease. We provide strong evidence that Myeloid differentiation factor-2 (MD2) plays a critical role in metastasis and CaP progression.

Analysis of tumor genomic data and IHC of tumors showed a high frequency of *MD2* amplification and association with poor overall survival in patients. The Decipher-genomic test validated the potential of *MD2* in predicting metastasis. *In vitro* studies demonstrated that MD2 confers invasiveness by activating MAPK and NF-kB signaling pathways. Furthermore, we show that metastatic cells release MD2 (sMD2). We measured serum-sMD2 in patients and found that the level is correlated to disease extent. We determined the significance of MD2 as a therapeutic target and found that targeting MD2 significantly inhibited metastasis in a murine model.

We conclude that MD2 predicts metastatic behavior and serum-MD2 is a non-invasive biomarker for tumor burden, whereas MD2 presence on prostate biopsy predicts adverse disease outcome. We suggest MD2-targeted therapies could be developed as potential treatments for aggressive metastatic disease.

## INTRODUCTION

Prostate cancer (CaP) is the most common cancer diagnosed in males, and is the second most common cause of cancer-related deaths [1]. Patients with localized CaP are generally curable. However, approximately 40% will experience a biochemical recurrence (BCR) within 10 years of treatment [2–5]. Furthermore, many of those who relapse will ultimately develop metastatic castration-resistant (mCRPC) disease [6], the principal cause of mortality [4, 5, 7–11]. Currently, there is no curative treatment for patients with metastasis. Therefore, understanding mechanisms that cause recurrence and confer metastatic characteristics to localized tumors would be essential for developing novel biomarkers of cancer progression and therapies to inhibit the disease progression.

Myeloid differentiation factor-2 (MD2) (*LY96*-gene) is a small glycoprotein expressed by macrophages and dendritic cells [12]. MD2 functions as a co-receptor for toll-like receptor (TLR) 4 and is required for its activation [13]. The TLR4 signaling pathway is involved in the oncogenesis of several cancers including CaP [14]. The expression of TLR4 and its activation are associated with CaP progression, therapy failure, and BCR [15, 16]. Since MD2 is essential in TLR4 signaling, targeting MD2 may be a potential therapeutic approach for treating patients. However, no studies have shown the expression or importance of MD2 in CaP.

In this study, we provide evidence that CaP cells produce and release MD2 during cancer progression, which results in activation of the MAPK and NF-kB signaling pathways. We also show that MD2 is an essential factor in the tumor microenvironment allowing CaP cells to acquire metastatic traits. Therefore, we speculate that *MD2*-addicted tumors are prone to metastasis. Aided by the Decipher-genomic test, we also provide evidence about the potential use of MD2 as a predictive biomarker of patient disease outcomes. In addition, we determined the significance of MD2 as a therapeutic using a mouse model of lung metastasis.

## MATERIALS AND METHODS

### Cell lines, antibodies, patient tissues, survival analysis, patient Cohort, transfections, and chemicals, Zebrafish in vivo model, lung metastasis model, and statistical analyses.

Descriptions are provided in Supplementary methods.

### Cell growth, migration, chemoinvasion and transmigration assays.

These assays were performed as described previously [17–19].

### Confocal microscopy, immunoblot, IHC, RT-qPCR, luciferase assay.

The experiments were performed per published methods [17, 18, 20, 21].

## RESULTS

### Alterations in the MD2 gene in CaP correlate with poor survival in patients

**Figure 1:**
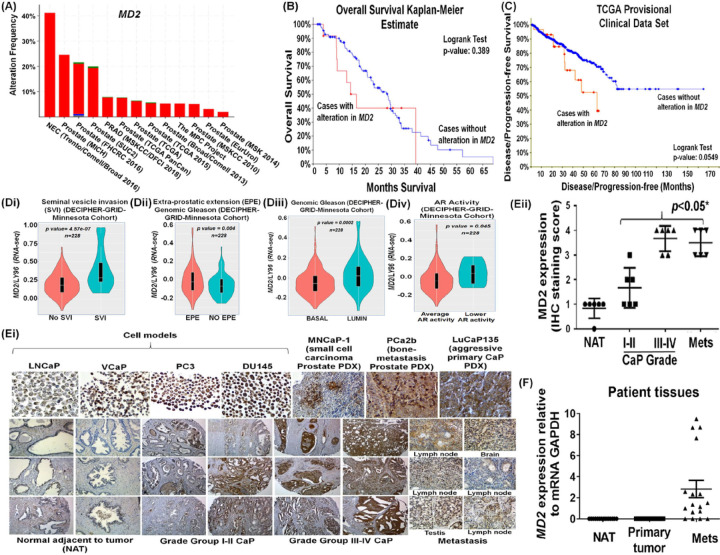
Relevance of MD2/LY96 alteration as a predictive biomarker of prostate cancer progression and poor survival in human patients. **(A)**The histogram shows the alteration frequency of the MD2/LY96 gene in 13 clinical studies comprised of 4,628 patients. The data shows the MD2 gene amplification (in red color) in the tumors of these patients. The data was analyzed from TCGA data sets. **(B)** Kaplan–Meier graph shows the analysis of TCGA clinical data establishing a correlation between MD2 expression and overall survival and **(C)** Disease progression-free survival in CaP patients. The data were generated from tumor genome analysis of patients using the cBioportal platform. Decipher genomic test. The graph shows the potential of the biopsy-MD2 alteration as a biomarker predicting the risk of seminal vesicle invasion **(Di)**, Extra-prostatic extension **(Dii)**, Genomic Gleason **(Diii)**, and AR activity **(Div)** by prostate tumor cells in CaP patients. Immunohistochemistry. **(Ei)** shows the expression of MD2 in prostate cancer cell-based models, patient-derived xenografts (PDX) models, one bone-metastasis cell-derived tumor xenograft and normal regions adjacent to tumor (NAT), primary prostate tumors with different grade groups, and metastatic tumors of CaP patients. **(Eii)** Staining score of the tissues assessed by IHC. **(F)**MD2/LY96 mRNA expression in patient tissues assessed by RT-qPCR. The data were normalized to GAPDH. The expression data is presented as a fold-unit change.

Since there is a strong association between cancer progression and inflammation, we asked if MD2 is involved in CaP; therefore, we studied the association of the presence of *MD2* to the survival of patients in a large patient cohort, with survival and follow-up details available. We performed a comprehensive analysis of the tumor genome data of patients using the cBioPortal web-platform. First, the genomic analysis of tumors of 4,628 CaP patients from 13 clinical studies suggested that the *MD2 gene* exhibits a high frequency of alterations, particularly amplification (40% cases) in neuroendocrine (NEPC) and castration-resistant (CRPC) cases (Fig. 1A). Then, we analyzed the overall survival of patients with *MD2* alterations. This clinical study (Metastatic Prostate Adenocarcinoma (SU2C/PCF Dream Team, PNAS) showed that cases with *MD2* gene alterations tend to exhibit a worse overall survival (Log-rank Test *p = 0.389*) than cases who have no alterations (Fig. 1B). The median overall survival for *MD2* altered patients was calculated as 14.5 months, whereas subjects without alterations exhibited an overall survival of 39.5 months (Fig. 1B). Next, we analyzed the disease/progression-free survival in the TCGA provisional clinical data set. The clinical study showed that patients with alterations exhibit significantly lower disease/progression-free survival than patients without alterations (Log-rank Test *p = 0.0549*) (Fig. 1C). Thus, survival data sets indicate that *MD2* amplification is associated with poor survival in patients. The *MD2* genetic alteration, therefore, has the potential to serve as a biopsy biomarker of poor prognosis and a risk factor for development of metastasis and shorter mortality. In the subsequent experiments, we studied the relevance of MD2 in CaP.

### MD2 as a predictive biomarker

Decipher test provides multiple algorithms to predict the clinical outcomes of CaP patients based on genome data of primary tumors. Based on a 22-gene signature, the basic Decipher test classifies patients as low, average, and high for therapy outcome or risk of recurrence or metastasis [22]. The predictive accuracy of the test can be made more robust by adding new algorithms such as the Genomic Gleason and CAPRAS algorithms; we asked if MD2 as a marker could identify locally invasive tumors prone to recur. We previously reported utilizing the Decipher test in a cohort of 228 patients that biopsy-S100A4 overexpression predicts poor ADT response and a high risk of mortality [23]. We used the same data cohort to study the association of MD2 with CaP progression, where the patients were classified as low, average, or high by the test. Seminal vesicle invasion (SVI) and extraprostatic extension (EPE) are validated indicators of poor outcomes and adverse prognosis in patients [24–26]. SVI is associated with increased likelihood of local recurrence and development of future metastasis and upstages prostate cancer to stage III CaP [27]. Our exploratory cohort was classified as “No SVI” and “SVI” by the test. Our data showed that *MD2*-high expression is significantly correlated to SVI-positive cases. We found that *MD2*-high expression cases are significantly (P = 4.57e^−07^) identifiable with Decipher-classified SVI cases (Fig. 1Di).

Extraprostatic extension (EPE) describes a tumor stage where the tumor extends beyond the prostate borders and is also associated with increased risk of metastasis [25, 28]. Patients in whom EPE is detected on prostate biopsy are considered to have adverse pathologic finding after RP [25]. The Decipher-algorithm predicts the risk of EPE. The test identified EPE cases in the cohort and classified them as EPE positive and NO EPE. When these cases were tested for *MD2* expression, a significant correlation (*p = 0.0004*) between high-*MD2* expression with EPE positivity was observed (Fig. 1Dii). These data support the notion that increased expression of *MD2* is associated with pathologic features of localized tumors which have increased risk for local and distant recurrence.

Decipher has developed multiple other cancer-subtype algorithms, which allow for finding common markers between sub-types and metastasis. The two outputs of the algorithm are “basal” and “lumen,” thus determining if a protein’s sub-tissue distribution affects the metastasis. Most metastatic prostate tumors are from the luminal region in prostate. The Decipher test classified the patient cohort as basal and luminal. The analysis showed that high-*MD2* cases are significantly (*p = 0.0002*) predicted to be of luminal sub-type CaP, whereas low-*MD2* cases could be basal type (Fig. 1Diii). Thus, multiple algorithms suggested that the expression of *MD2* is associated with poor clinical outcomes.

Aggressive CaP cases with CRPC features can be of several sub-types, including those with either high-AR or low-AR activity [29, 30]. Indeed, CRPC disease with low-AR activity has been identified as highly aggressive and refractory to treatment. Decipher has algorithms based on gene signatures which classifies cases to some degree into low-AR and average-AR activity tumor subtypes. The patient cohort analysis showed that high-*MD2* expression is found in most low-AR activity CaP cases (*p = 0.045*), again indicating its association with the aggressiveness of disease (Fig. 1Div).

These findings in a Decipher cohort establish the correlation of MD2 with advanced disease and metastasis.

### MD2 protein levels in prostate tumors

Aggressive tumor cells in the tumor microenvironment (TME), while getting addicted to certain factors present in the TME, also start expressing such factors themselves. MD2 is mainly found in immune cells. However, its presence in prostate tumors has not yet been reported. Therefore, we performed the IHC analysis to determine the presence of MD2 in CaP tissues. First, we performed antibody validation and specificity testing by IHC of Histogel-embedded metastatic CaP (mCaP) cell models (LNCaP, VCaP, PC3, and DU145). We found that the antibody detected MD2 in CaP cells and differentiated between MD2-rich cells from the MD2-deficient cell model. This is evident from the data where MD2 was found to be highly expressed in VCaP, PC3, and DU145 whereas it is scantily present in LNCaP (Fig. 1Ei). It is to be noted that VCaP, PC3 (bone metastasis-derived), and DU145 (brain metastatic-tumor derived) are considered highly aggressive cells, whereas LNCaP is a slow-growing lymph-node-derived cell line. Second, we evaluated the expression of MD2 in two primary patient-derived xenografts (PDX) models and one bone-metastasis cell-derived tumor xenograft. These included LuCaP135 (primary invasive tumor), MNCaP1 (aggressive primary prostate small cell carcinoma), and PCa2b (PCa2b cell-derived bone tumor). The IHC confirmed the presence of MD2 in all PDX tumors (Fig. 1Ei).

Next, we evaluated the expression of MD2 in NAT (normal tissues adjacent to the cancerous region), primary (Grade Groups (GG) I-II or GG III-IV), and metastatic tumors (lymph-node, brain, testis). The IHC of patient specimens showed that prostatic tumors exhibit elevated immunostaining compared to the NAT (Fig. 1Ei). Notably, metastatic tumors exhibited more positive immunostaining for MD2 than primary tumors (Fig. 1Ei, Eii). The immunostaining intensity was scored on a scale of 0–4 (0 = none, 1 = weak/scant, 2 = moderate, 3 = strong, 4 = highly strong). Based on the immunostaining score, we compared the expression of MD2 between tumor grades. In comparison, MD2 expression in primary tumors of GG I-II (*p < 0.05)* and GG III-IV *(p < 0.001)* was significantly higher than NAT (Fig. 1Eii). The expression in GG-III/IV was almost equal to metastatic tumors (Fig. 1Eii). Out of all metastatic tumors, metastatic brain tumors exhibited higher MD2-immunopositive cells (Fig. 1Eii). These data show that MD2 is highly expressed in GGIII/IV primary and metastatic tumors and that MD2 levels increase progressively during disease progression.

We asked if the increment of the MD2 during CaP progression is a translational event or originates at the transcriptional level. For this, we performed qPCR analysis of human primary prostate tumors, metastatic tumors, and NAT. Metastatic tumors exhibited a higher level of *MD2* transcript than primary tumors (*p* < *0.05*). Notably, some metastatic tumors showed *MD2* like primary tumors (Fig. 1F). These data suggest that an increase in *MD2* occurs during progressive phases of CaP and suggest *MD2* as an indicator of disease progression in patients.

### MD2 expression in a cell-based progression model

**Figure 2:**
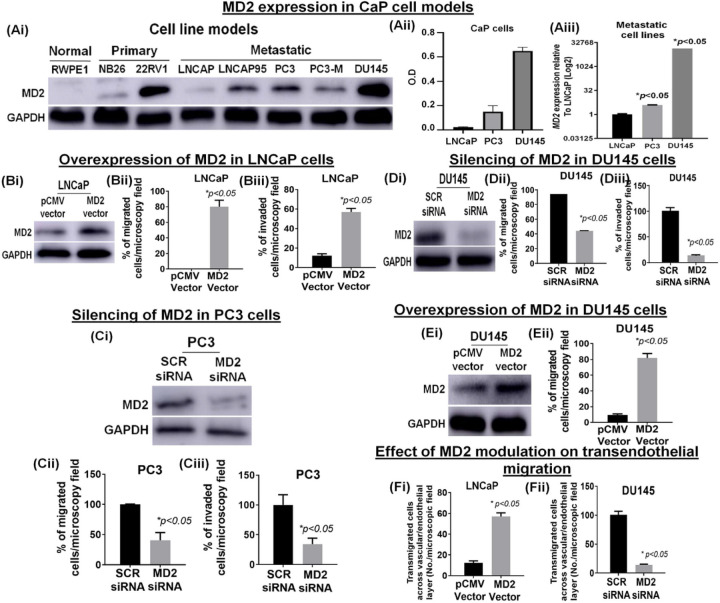
Relevance of MD2 for metastasis of the prostate cancer cell lines. **(Ai)**Immunoblot image shows the protein level of MD2 in CaP cell models representing normal (RWPE1), premalignant/indolent PCa (RW-NB26), primary PCa (22Rv1), lymph-node metastasis (LNCaP), AR-positive CRPC (LNCaP95), bone-metastasis (PC3 and PC3-M), and brain-metastasis (DU145), assessed by immunoblot analysis. The GAPDH protein levels in cell lysates were used as a loading control. **(Aii)**Quantification of MD2 by densitometry in LNCaP, PC3, and DU145 cells. **(Aiii)**Expression of mRNA MD2 in LNCaP, PC3, and DU145 cells assessed by RT-qPCR. **(Bi)**Immunoblot image shows the expression of MD2 in LNCaP expressing the MD2 vector. **(Bii-iii)** Histograms compare the migratory **(Bii)** and invasive **(Biii)** potential of LNCaP cells expressing the MD2 vector. **(Ci, Di)** Immunoblot images show the effect of MD2-suppression (by siRNA transfection) on MD2 protein in PC3 and DU145 cells. **(Cii-iii, Dii-iii)** Histograms compare the migratory **(Cii, Dii)** and invasive **(Ciii, Diii)**potential of PC3 and DU145 cells silencing MD2**. (Ei)** Immunoblot image shows the overexpression of MD2 in DU145 cells and its effect on migration **(Eii). (Fi-ii)** Bar graphs show the effect of MD2 overexpression (LNCaP) or silencing (DU145) on the transendothelial migration through a layer of endothelial cells (HUVEC).

Although we showed the expression of MD2 in a few CaP cell models (Fig. 1Ei), we expanded our examination to a spectrum of CaP cell lines. We evaluated the level of MD2 in normal (RWPE1), primary indolent (NB26), primary-CRPC (22RV1), androgen-dependent metastatic (LNCaP), and mCRPC (LNCaP95, PC3, PC3-M, and DU145) models by immunoblotting. The results showed that MD2 is significantly high in CRPC cells, particularly in metastatic cells (Fig. 2Ai). The exception was LNCaP which exhibited scant expression of MD2, which corroborated with our IHC data (Fig. 1Ei). The analysis by densitometry shows an expression level in DU145 > PC3 > LNCaP (Fig. 2Aii). Next, we compared the *MD2* transcript in DU145, PC3, and LNCaP cells and found that cells with high MD2 protein harbor a high level of *MD2* transcript *(p < 0.05)* (Fig. 2Aiii). These data show that MD2 is associated with advanced CaP in a progressive cell model and further cements the position of MD2 as a potential biomarker of CaP progression and metastasis.

### Intracellular expression of MD2 induces migration and invasion

Since LNCaP exhibit a lower expression of MD2 and a lower metastatic potential than PC3, and DU145, we evaluated the significance of the MD2 in these metastatic cells. Thus, to study the role of MD2 in CaP progression, we focused on LNCaP, PC3, and DU145. To investigate the role of MD2 in metastasis, we overexpressed MD2 in LNCaP. We confirmed the overexpression by immunoblotting (Fig.2Bi) and immunofluorescence **(Supplemental Figure Ai)** and evaluated the viability, migration, and invasion. We found that the ectopic overexpression of MD2 did not alter the proliferation rate assessed by MTT and by immunoblotting, evidenced by the expression of PCNA **(Supplemental Figure Aii, iii)**. However, MD2 regulated the migration and invasion of these cells. The overexpression of MD2 results in an enhanced migration and invasion compared with controls (Fig. 2Bii-iii). Then, we silenced MD2 in PC3 and DU145 (MD2 siRNA) (Fig. 2Ci, Di) and evaluated the viability, migration, and invasion. As expected, the silencing did not modify the cell growth **(Supplemental Figure Aiv, v)**. However, the silencing inhibited migration and invasion (Fig. 2Cii-iii, Dii-iii). We also overexpressed MD2 in DU145 cells (Fig. 2Ei), which exhibit a high basal level of MD2, and then assessed migration. The result showed that DU145 overexpressing MD2 display a higher rate of migration than the control (Fig. 2Eii) **(Supplemental Figure B)**. Finally, we evaluate the effect of the modulation of MD2 in LNCaP and DU145 on the potential of the cells to cross the vascular barrier (layer of human endothelial cells (HUVEC)) using an *in vitro* model of extravasation. The overexpression of MD2 in LNCaP induced a significant increment in the number of transmigrated cells (Fig. 2Fi), and to the contrary, silencing of MD2 in DU145 caused a significant decrease (Fig. 2Fii). These results suggest MD2 confers metastatic behavior characterized by increased migration and invasion.

### Intracellular expression of MD2 induces NF-kB signaling

**Figure 3:**
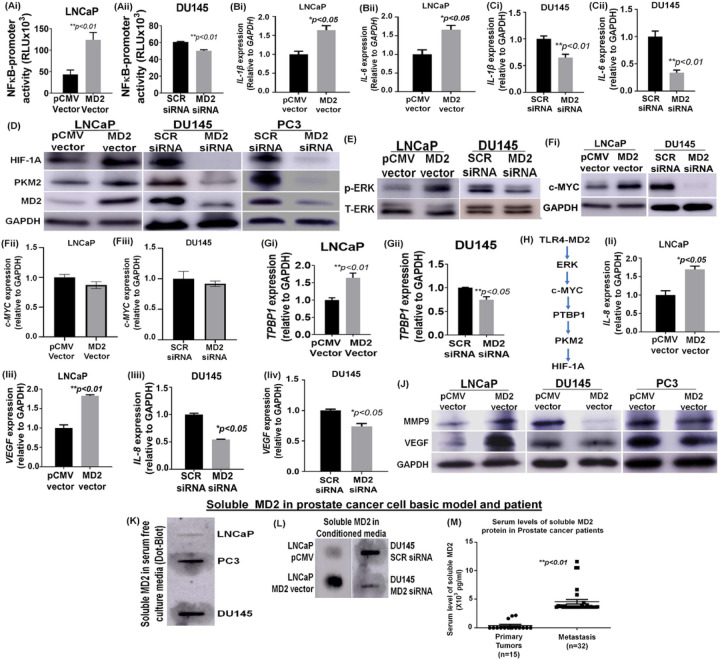
MD2 signaling pathways and soluble MD2. **(Ai-ii)**Histograms compare the NF-kB transcriptional activity in LNCaP overexpressing MD2 and DU145 silencing MD2 assessed by dual-luciferase reporter assays. Each bar of histograms represents the average of three independent experiments. Renilla luciferase activity served as the internal control for each group. **(Bi-ii, Ci-ii)** Histograms show the effect of the MD2 vector in LNCaP cells or MD2 siRNA in DU145 cells on IL-1B and IL-6 mRNA assessed by RT-qPCR. **(D)**Immunoblot images show the effect of MD2 overexpression (LNCaP) or MD2 suppression (DU145 and PC3) on HIF-1A and PKM2 expression. **(E)**Immunoblot images show the effect of MD2 overexpression or suppression on phospho- and total-ERK proteins. **(Fi-iii, Gi-ii)** Effect of MD2 overexpression or suppression on c-MYC protein assessed by immunoblotting and c-MYC and TPBP1 mRNA assessed by RT-qPCR. **(H)** MD2 downstream targets model suggested. **(Ii-iv)** Histograms show the effect of the MD2 vector in LNCaP cells or MD2 siRNA in DU145 cells on IL-8 and VEGF mRNA assessed by RT-qPCR. **(J)** Immunoblot images show the effect of MD2 overexpression (LNCaP) or suppression (DU145 and PC3) on MMP9 and VEGF proteins. **(K, L)**Dot blot images show the absence or presence of soluble MD2 in conditioned media of LNCaP, PC3, and DU145 cells and the effect of MD2 overexpression or suppression on soluble MD2. **(M)** Serum levels of soluble MD2 in prostate cancer patients diagnosed with primary tumors or metastasis assessed by ELISA.

To explore the mechanisms underlying the MD2-dependent metastatic observation we focused on the TLR4 signaling pathway because MD2 is required for its activation. Thus, we measured the NF-kB promoter activity in LNCaP overexpressing MD2 and DU145 silencing MD2. We found that the overexpression of MD2 induced strong activation of the NF-kB promoter activity (Fig. 3Ai), and to the contrary, silencing of MD2 in DU145 resulted in a lower level of activity (Fig. 3Aii). In addition, we assessed the expression of p65, which is increased in cells with sustained activation of NF-kB signaling. We found that in cells overexpressing MD2, p65 was higher than the control. Contrarily, cells silencing MD2 exhibit a lower expression **(Supplemental Figure Ci)**.

Then, we evaluated the expression of the downstream targets of TLR4, namely IL-1β, and IL-6 by qPCR. We found that LNCaP overexpressing MD2 exhibit high levels of both interleukins (Fig. 3Bi, ii). We also evaluated the expression of TNF-α and IL-10, which have been described to be induced by TLR4 activation in macrophages and monocytes [31], but we did not find any difference compared with the control cells (pCMV vector) **(Supplemental Figure Cii, iii)**. On the contrary, the silencing of MD2 in DU145 resulted in a lower expression of IL-1β and IL-6 (Fig. 3Ci, ii). These data show that the expression of MD2 in metastatic CaP cells induces pro-inflammatory cytokines through the activation of NF-kB signaling.

### Intracellular expression of MD2 regulates HIF-1A and PKM2

The activation of TLR4 by some ligands results in the induction of HIF-1A and PKM2 via the activation of NF-kB in macrophages [32]. However, the consequences of its activation in CaP cells are unknown. Therefore, we evaluated if the expression of MD2 induces HIF-1A and PKM2 in CaP cells. For this, we assessed the levels of HIF-1A and PKM2 by qPCR and immunoblotting in cells overexpressing and silencing MD2. The results showed that overexpression or silencing of MD2 did not modify the levels of these mRNAs **(Supplemental Figure Di-iv)**; however, we found a significant change in the protein levels. LNCaP overexpressing MD2 exhibit a strong expression of HIF-1A and PKM2 (Fig. 3D), while silencing of MD2 in PC3 and DU145 resulted in a marked decrease of the expression of both proteins (Fig. 3D). It is well documented that HIF-1A and PKM2 exhibit positive feedback, and PKM2 can bind HIF-1A protein inducing its stabilization [33]. Therefore, we investigated upstream PKM2 proteins. Since activation of TLR4 results in ERK phosphorylation that induces stabilization of c-MYC, we evaluated phospho-ERK in cells overexpressing and silencing MD2 and its possible role in c-MYC stabilization. We found that LNCaP overexpressing MD2 exhibit strong phosphorylation of ERK (Fig. 3E). On the contrary, silencing MD2 in DU145 decreased the phosphorylation (Fig. 3E). Furthermore, we found that the high level of phospho-ERK was associated with high levels of c-MYC protein. However, we did not find any change in the mRNA expression levels (Fig. 3Fi-iii). c-MYC induces the expression of PTBP1, which causes the switch from PKM1 to PKM2 [34]. Therefore, we assessed the levels of *PTBP1* by qPCR. The result showed that cells overexpressing MD2 exhibit a significant increment of *PTBP1* (Fig. 3Gi), while cells silencing MD2 exhibit lower levels (Fig. 3Gii). These data suggest that MD2 induces activation of ERK and stabilizes c-MYC at the protein level, which in turn may induce an increment in the level of PKM2 protein via induction of PTBP1, leading to HIF-1A stabilization (Fig. 3H).

### Intracellular expression of MD2 induces IL-8 and VEGF.

IL-8 expressed by CaP cells promotes metastasis and aggressiveness [35]. Since it was reported that HIF-1A mediates the induction of IL-8 and VEGF [36], we evaluated if the overexpression of MD2 results in higher levels of IL-8 and VEGF. Therefore, we assessed the levels of *IL-8* and *VEGF* transcripts in cells overexpressing and silencing MD2. The results showed that LNCaP overexpressing MD2 exhibited a high level of *IL-8* and *VEGF*, while silencing of MD2 in DU145 resulted in a decrease in the level of these transcripts (Fig. 3Ii-Iiv). These data suggest that MD2 may regulate the expression of *IL-8* and *VEGF* in metastatic CaP cells, possibly through the induction of HIF1-A.

### Intracellular expression of MD2 induces VEGF and MMP9

Due to mCaP cells expressing high levels of MMP9 required for metastasis and VEGF participating in a positive feedback regulation between MMP9 and VEGF, we evaluated the effect of MD2 on the expression of MMP9 and VEGF by immunoblotting. The results showed that the overexpression of MD2 in LNCaP induced a significant increment of MMP9 and VEGF. To the contrary, in PC3 and DU145 cells, MD2 silencing resulted in levels of MMP9 and VEGF that were lower than controls (Fig. 3J). All these data show that MD2 regulates several signaling pathways involved in metastasis and confers aggressive characteristics to CaP cells.

### Soluble MD2 as a new biomarker for advanced CaP

To study MD2 as a potential biomarker, we asked if mCaP cells expressing MD2 release the soluble form (sMD2) into the microenvironment. We first assessed the localization of MD2 in LNCaP overexpressing MD2 by IHC. We found that in LNCaP, the expression was very poor, and the localization was focal in the interior of the cells. In contrast, in LNCaP overexpressing MD2, the expression was very intense. The distribution was focal in the interior of the cells, with robust staining in the membrane and outside the cells **(Supplemental Figure E)**, which suggests that MD2 may be released outside the cells. To evaluate this possibility, we assessed the presence of sMD2 in the conditioned media from LNCaP, PC3, and DU145 by dot-blot. We found that sMD2 was detected only in highly metastatic cells (PC3 and DU145) (Fig. 3K). Then, we evaluated the effect of overexpressing and silencing MD2 in LNCaP and DU145 on the release of sMD2. For this, we measured sMD2 in conditioned media from stables clones of LNCaP (pCMV and MD2 vector) and DU145 (DU145 SCR siRNA and MD2 siRNA). We found that sMD2 was detected in conditioned media from LNCaP overexpressing MD2 but not in the control (pCMV) (Fig. 3L). Conversely, the silencing of MD2 in DU145 resulted in a lower level of sMD2 in the conditioned media (Fig. 3L). Then, we evaluated sMD2 as a biomarker for advanced CaP. Therefore, we measured sMD2 in the serum samples of patients diagnosed with CaP by ELISA. We included 15 primary tumors and 32 metastatic tumors. The results showed that the levels of sMD2 correlated with the progression of the disease. Metastatic patients exhibit a higher level of sMD2 with a mean of 4.5 pg/ml (3.6–11.6 pg/ml) compared with patients with primary tumors with a mean of 0.53 pg/ml) (0–2.2 pg/ml) (**p < 0.01) (Fig. 3M), with most of them displaying an undectecable level of sMD2. Thus, these data strongly suggest that sMD2 may be used as a novel biomarker for advanced CaP disease.

### Cancer cell extravasation model of metastasis in zebrafish

**Figure 4:**
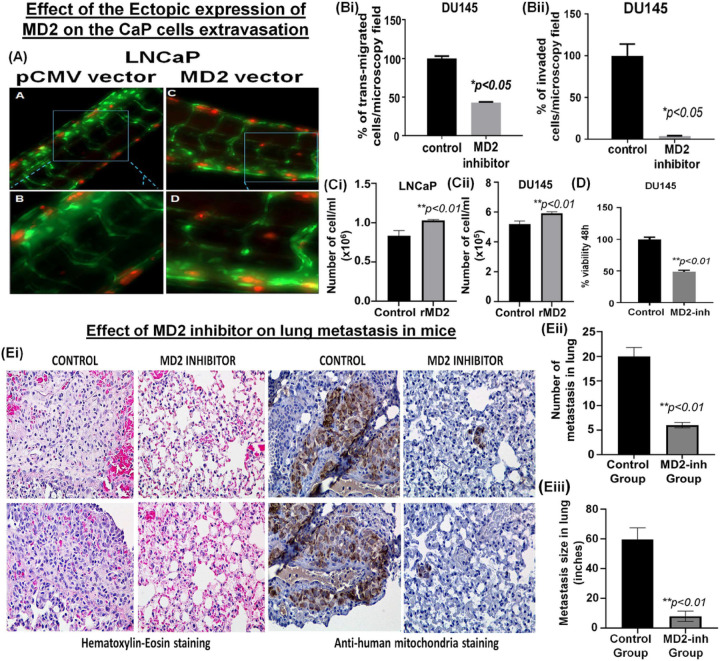
Targeting MD2 as a therapeutic approach. **(A)**Image shows the effect of overexpressing MD2 on the LNCaP cells extravasation in a Zebrafish in vivo model for metastasis. LNCaP cells were transiently labeled with a fluorescent tracker dye (red), and the bloodstream is in green (GFP) **(Bi-ii)** Bar graphs show the effect of the pharmacological targeting MD2 with a small molecule inhibitor (10 μM) in DU145 cells on transmigration and invasion. **(Ci-ii)** Bar graphs show the effect of recombinant human MD2 protein on proliferation assessed by cell counting. **(D)** The bar graph shows the effect of MD2 inhibitor (10 μM) on cell viability assessed in 48h by MTT assay. **(Ei**) Effect of targeting MD2 in a mouse lung metastasis model. Images compared the hematoxylin-eosin and anti-mitochondria lung tissue staining in control and treated (MD2 inhibitor) mice. **(Eii-iii)**Bar graphs show the number and size of metastasis in control and treated mice.

During metastasis, tumor cells originating from the primary site migrate through the bloodstream and colonize distant locations. As part of this process, tumor cells invade and reside in non-primary sites by extravasating from the bloodstream. Zebrafish have emerged as an important vertebrate model to study cancer metastasis because they are amenable to *in vivo* imaging and share histological and genetic similarities with humans [37–40]. We have previously established a zebrafish xenotransplantation model of human cancer cell extravasation, which enables the visualization and assessment of extravasation events [41–43]. To assess the effect of MD2 in the extravasation and metastasis of human CaP cells, LNCaP stably overexpressing MD2 (and control) were transiently labeled with a fluorescent tracker dye and microinjected into the bloodstream 3 days post-fertilization Tg(fli:GFP) of zebrafish embryos via the pericardium. The following day at 24 hours post-injection of the cells, the larval zebrafish were imaged using a fluorescence microscope. We found that control LNCaP cells remained in the vasculature, whereas LNCaP stably overexpressing MD2 were in the extravascular space (Fig. 4A, **Supplemental Figure F)**. These *in vivo* results suggest that MD2 promotes the extravasation of human CaP cells, an important step required by metastasis.

### Inhibition of MD2 suppresses lung metastasis in a murine model

Since ectopic expression of MD2 in LNCaP resulted in increased extravasation, we studied the effect of the inhibition of MD2 in DU145 on the transmigration ability through HUVEC and chemoinvasion. We treated DU145 with MD2-int-1, a small molecule inhibitor of MD2, and evaluated the transendothelial migration and invasion in 24h. The results showed that the treatment significantly decreased both metastatic characteristics (Fig. 4Bi, Bii). These results reinforce our hypothesis that MD2 is involved in metastasis in CaP for promoting and facilitating migration and invasion of the cancer cells.

Because the inhibitor suppresses the transendothelial migration ability of the metastatic cells led us to test the inhibitory effect of the inhibitor in a mouse model of lung metastasis. First, we evaluate the effect of recombinant MD2 (rMD2) on cell growth in LNCaP and DU145. As shown (Fig. 4Ci, ii), rMD2 induced a moderate but significant increment in the number of cells assessed by counting. Then, we treated DU145 with the inhibitor and evaluated the viability by MTT. The results show that treatment resulted in a reduced percentage of viable cells compared with the control (Fig. 4D). Finally, we tested the efficacy of the therapy in lung metastasis in mice. We used DU145 cells that develop lung metastasis after 15 days of tail vein injection. After treatment, the mice were euthanized, and the lugs were dissected and analyzed by Hematoxylin-eosin staining and IHC. As shown (Fig. 4Ei), the hematoxylin-eosin staining shows that the treatment inhibited almost absolutely the presence of lung metastasis.

In comparison to the treated group, the control group exhibited substantial infiltration of tumor cells, which results in the loss of the normal shape and characteristics of the lung (Fig. 4Ei). Furthermore, when we compared the number of metastases in both groups, we found that the group treated with MD2 inhibitor showed an average of 5 metastasis, while in the control group, the average of metastasis was 20 (Fig. 4Eii). In addition, we evaluated the presence of human mitochondria in the lungs by IHC. The result showed intense staining in lungs obtained from the control group, while a small focus was observed in the treated group (Fig. 4Ei). Furthermore, the measure of the metastasis size in both groups was significantly different; while the average size in the control group was around 60 inches, in the treated group was 8 inches (Fig. 4Eiii). All these data show that MD2 actively participates in lung metastasis in mice and is a druggable target to treat metastatic CaP.

## DISCUSSION

The high rate of recurrence and metastasis in CaP, and the lack of curative therapy for this, highlight the need to develop new and more efficient therapies [4, 5, 7–11]. Therefore, it is imperative to identify novels therapeutic targets and new biomarkers for cancer progression. It is obviously recognized that patients with localized CaP often respond to primary therapy; however, a substantial number of men will acquire resistance and develop metastasis [4, 5, 7]. Identifying novel biomarkers associated with CaP progression will help the clinician better guide therapy, perhaps choosing adjuvant therapies post initial local therapy, and to better monitor/evaluate the progression of the treatment. Since BCR is observed in up to 40% of patients [4, 5, 7–9], we need to identify novel biomarkers to evaluate the response to the treatment and disease progression before the BCR. This study assesses the significance of MD2 detection in patients and its role in CaP.

Since we found a strong association between the expression of MD2 and metastasis, we analyzed the significance of MD2 in the outcome of the patients by the analysis of a large patient cohort (TCGA-PRAD), where we found alterations in *MD2* are associated with the poor outcome. Therefore, the clinical data strengthens our hypothesis that MD2 is involved in aggressiveness, metastases, and CaP progression.

The current study delves into the role of MD2 as a predictive biomarker of metastasis and cancer progression in patients who undergo primary treatment after RP. The Decipher test is used to predict the outcome of the patients. Thus, by employing the test, we successfully validated the significance of the expression of *MD2* in patients who were predicted to develop metastasis and the worst outcome. Interestingly, the expression of *MD2* in these patients predicted a low level of AR activity; our interpretation is that metastatic cells expressing high levels of MD2 do not require high levels of AR activity to grow. We posit that metastatic cells expressing MD2, and low AR activity will be resistant to traditional anti-AR therapies. Given that there is no reliable biomarker of the disease progression, the determination of MD2 in the biopsy of the patients should be evaluated in more studies to confirm our suggestion.

We provide strong evidence that metastatic cells express and release MD2 during cancer progression providing the cell with increased migration and invasiveness potential. The metastatic characteristics were associated with the activation of the MAPK and NF-ΚB signaling pathways.

On the other hand, by using an *in vivo* extravasation model, we provide evidence that the expression of MD2 induces transendothelial tumor cell migration, which is essential for developing metastasis. Furthermore, in a lung metastasis model we were able to show that MD2 is a druggable target for metastatic CaP. Therefore, MD2-targeted therapies, or therapies directed at its downstream protein products, could be developed as potential treatments for aggressive metastatic CaP.

Finally, the high levels of sMD2 in CaP patients correlate with the progression of the disease, highlighting the need to determine sMD2 in patients due to represent a potential non-invasive biomarker for the advancement of the disease and treatment failure.

In conclusion, MD2 expression on biopsy tissue would improve the performance of the Decipher-test in predicting the disease outcome in CaP patients. MD2 represents a potential non-invasive new biomarker for advanced CaP, and detection in both biopsy specimens and serum may allow treating the patients with more aggressive early therapy to improve the outcomes and treat micrometastatic disease. Furthermore, therapies targeting MD2 may potentially treat aggressive CaP. For clinical use, MD2-targeting agents warrant a thorough investigation in animal models of CaP.
